# The diagnosis and treatment of a special rare type of Monteggia equivalent fractures in children

**DOI:** 10.3389/fped.2023.1120256

**Published:** 2023-03-28

**Authors:** Fei Su, Min Li, Yishan Ma, Yating Yang, Xue Hao, Haoruo Jia, Youting Dang, Qingda Lu, Chenxin Liu, Shuai Yang, Huan Wang, Bing Wang, Qiang Jie

**Affiliations:** ^1^ Department of Pediatric Orthopedics, Honghui Hospital, Xi’an Jiaotong University, Xi’an, China; ^2^ Research Center for Skeletal Developmental Deformity and Injury Repair, School of Life Science and Medicine, Northwest University, Xi’an, China; ^3^ Clinical Research Center for Pediatric Skeletal Deformity and Injury of Shaanxi Province, Xi’an, China

**Keywords:** Monteggia equivalent, radial neck, fixation, children, diagnosis and treatment

## Abstract

**Purpose:**

To explore the characteristics, mechanism, treatment, and prognosis of head–neck separation type of Monteggia equivalent fractures in children.

**Methods:**

Patients with this injury were reviewed retrospectively. The lesion was characterized by a fracture of the ulnar with radial neck fracture but without dislocation of the radial head. Our classification was based on the direction of displacement and angulation of fractures on radiographs, divided into the extension-valgus type and flexion-varus type. The fractures were treated with reduction and internal fixation, depending on the fracture type. The clinical results were evaluated by using radiology and the Mayo Elbow Performance Score (MEPS).

**Results:**

A total of 12 patients were followed up for an average of 40.5 months. The ulnar fractures were treated with closed reduction (CR) and K-wire fixation in one patient, elastic stable intramedullary nail (ESIN) fixation in four patients, open reduction (OR) and plate fixation in five, with no fixation in two. CR with ESIN fixation was successful in 11 patients with radial neck fractures, but one underwent OR and K-wire fixation. All fractures healed on time, with fewer complications (avascular necrosis in one patient, and bulk formation of metaphysis in another). The therapeutic efficacy was evaluated by using MEPS and was found to be excellent in 10 patients, good in one, and fair in another.

**Conclusions:**

The head–neck separation type of Monteggia equivalent fractures in children is rare. Its characteristics are different from that of Monteggia fracture. The length and anatomic structure of the ulna should be restored and stabilized first, while the radial neck fracture should be treated with CR and ESIN fixation. Satisfactory clinical results can be achieved with fewer complications.

## 1. Introduction

Monteggia fracture, named after Giovanni Monteggia in 1814, and well-described and classified by Dr. Bado in 1967 (1), involves ulnar fracture and a concomitant dislocation of the radial head. The term “lesion” has gradually superseded those such as “fracture,” “fracture–dislocation,” or “injury” in the literature, stressing the importance of noticing the radiocapitellar joint and reflecting an increased awareness of the complexity regarding its manifestation and mechanism among orthopedists. The groups of “Monteggia equivalent lesion/variant” have considerably expanded after decades of reports of sporadic cases, apart from the established four types proposed by Bado (1). The boundary of that definition has blurred to a great extent. Also, especially in pediatric patients, when immature radiocapitellar epiphysis interferes with judgment and the flexible joint allows more frequent subluxation, a large number of these types tend to be misdiagnosed or neglected because of the occult presentation of the radiocapitellar joint or plastic bowing ulna on radiographs. The Monteggia equivalent fractures proposed by Bado (1) refer to injuries that share similar mechanisms, imaging manifestations, and treatment principles with Monteggia fractures, mainly including Bado type I and type II. However, most of the Monteggia equivalent fractures do not lead to a separation of the proximal radioulnar joint, which is the main difference between Monteggia fractures and Monteggia equivalent fractures. This article aims to describe a special rare type of Monteggia equivalent fractures in children, called head–neck separation type. At present, there are only three case reports on this injury in children (2–4), with no understanding and research on its characteristics or treatment principles. Here, we reviewed 12 patients, the maximum number of such cases at present, diagnosed as a head–neck separation type of Monteggia equivalent fracture in our department from March 2016 to February 2019. By summarizing and analyzing the clinical characteristics, treatment, and prognosis, we hope that our effort will have clinical significance and prevent the risks of misdiagnosis and improper treatment and also enhance the understanding of the concept and clinical classification of Monteggia equivalent fractures in children.

## 2. Materials and methods

### 2.1. Ethical consideration

This study was approved by the Ethics Committee of Hong Hui hospital, Xi'an Jiaotong University. All guardians of the minors provided written informed consent prior to participation in the study. All methods were carried out in accordance with relevant guidelines and regulations (Declaration of Helsinki).

### 2.2. Patient selection

Inclusion criteria: (1) children aged 0–15 years; (2) children with a fracture of the ulnar diaphysis or metaphysis; (3) children with a separated fracture of the radial neck without dislocation of the radial head; and (4) children that could be followed up completely.

Exclusion criteria: (1) older than 15 years; (2) multiple fractures; (3) open fractures; (4) children needing surgical exploration because of neurovascular injuries; and (5) incomplete clinical data.

A total of 12 patients who were examined and treated in our department for head–neck separation type of Monteggia equivalent fractures from March 2016 to February 2019 were identified, and their medical records and radiographs were reviewed retrospectively. There were eight boys and four girls with an average age of 8.3 years (range, 3–14 years). The left, non-dominant limb was involved in nine patients, and the right, dominant extremity was involved in three. The causes of injury were falling from a scooter (six patients), falling from horizontal bars (three), a traffic accident (two), and falling from a cycle (one). All fractures were closed, with a mean time of 9.2 h (range, 2–24 h) from injury to consultation.

The clinical manifestations were significant pain with a deformity of the forearm and significant swelling. Physical examination showed that one of the patients could not bend and stretch the elbow actively, with limited movement due to pain. The skin was intact, and the passive finger-pulling pain was negative, but there was a radial nerve injury in one patient.

**Figure 1:**
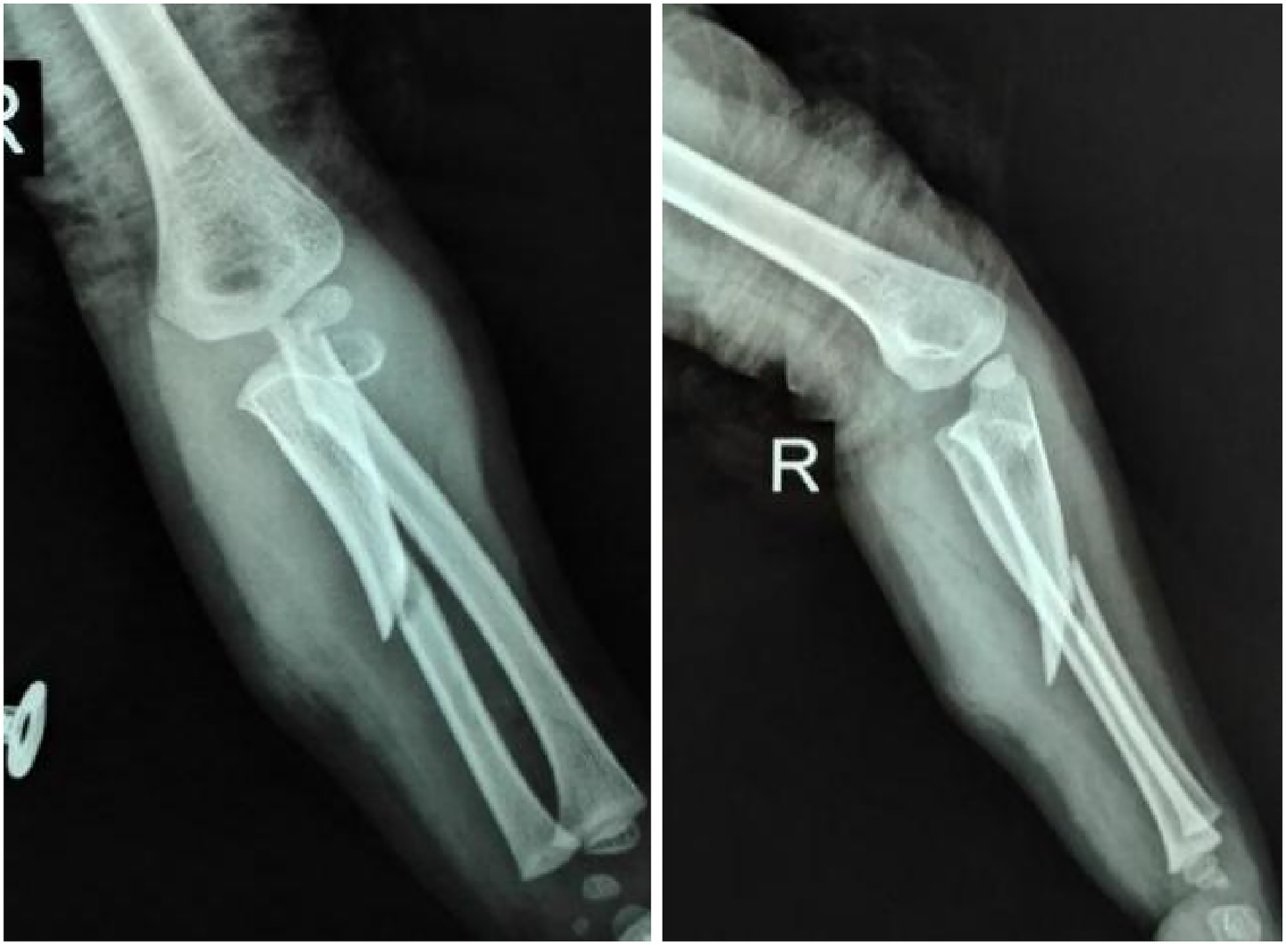
Extension-valgus type, an ulnar fracture with volar ulnar angulation plus a radial neck fracture with a volar ulnar displacement of the distal end.

**Figure 2:**
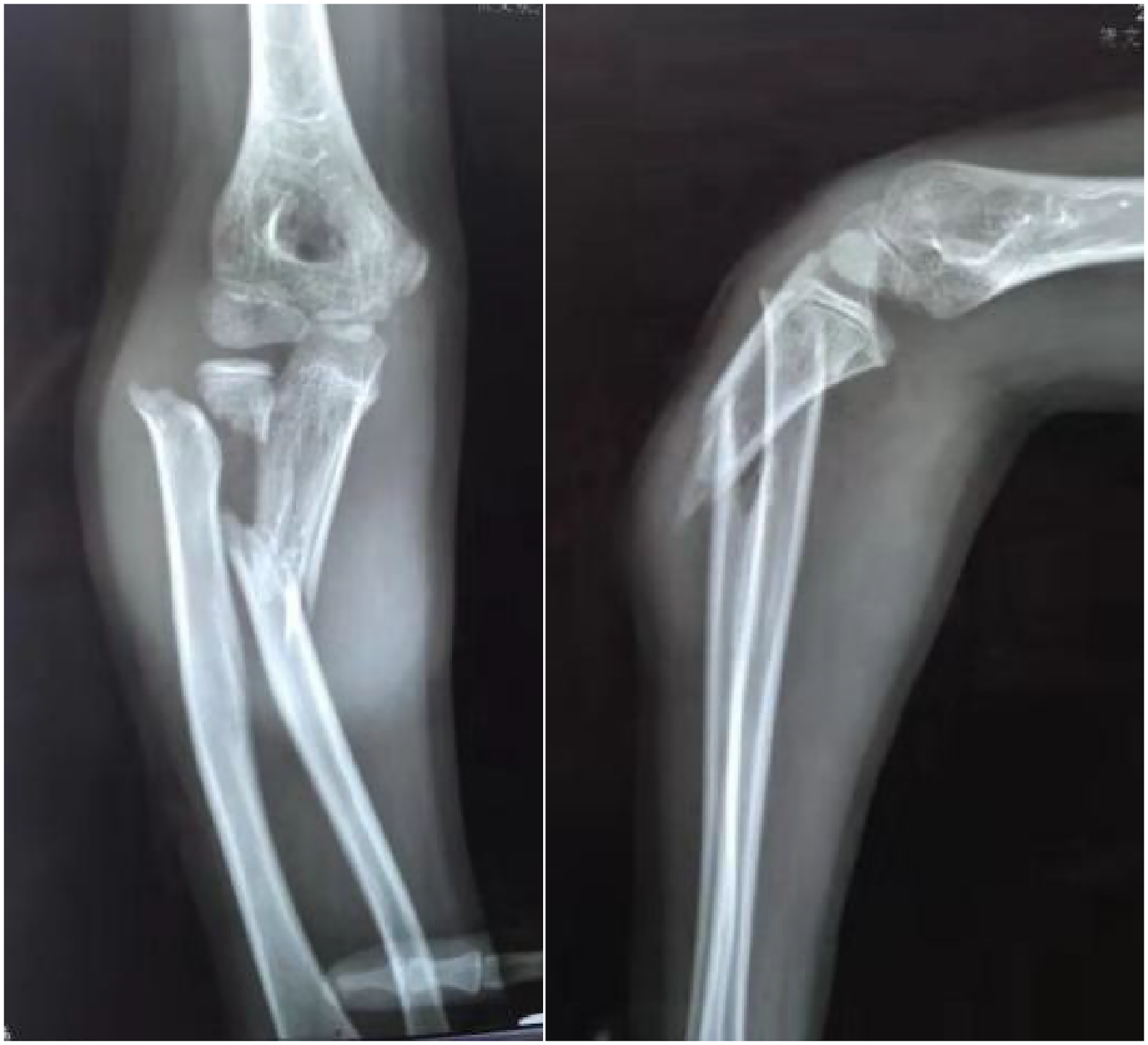
Flexion-varus type, an ulnar fracture with radial dorsal angulation plus a radial neck fracture with a radial dorsal displacement of the distal end.

By analyzing and summarizing these patient cases, we based our classification on the direction of displacement and angulation of fractures on radiographs. Seven cases belonged to the extension-valgus type with an ulnar fracture with volar ulnar angulation, plus radial neck fracture with a volar ulnar displacement of the distal end (Figure 1). Five cases were classified as the flexion-varus type with an ulnar fracture with radial dorsal angulation, plus radial neck fracture with radial dorsal displacement of the distal end (Figure 2).

According to the ulnar fracture sites, there were three patients with a metaphysis fracture (one case of the extension-valgus type and two of the flexion-varus type), seven patients with a proximal third ulnar fracture (five cases of the extension-valgus type, and two of the flexion-varus type), and two patients with a middle third ulnar fracture (one case of the extension-valgus type and one of the flexion-varus type). All the ulna metaphysis fractures were greenstick, with little displacement in one patient and obvious displacement and longitudinal splitting in two others. The angulation of the proximal and middle third ulna fractures was obvious, characterized by oblique fractures (six cases of short oblique and three cases of long oblique), with some fractures accompanied by vertical splitting at the proximal end. All the radial neck fractures were located in the metaphysis, without involving the proximal epiphysis and epiphyseal plate, and at the same time, all the proximal radioulnar joints were normal. This was in total contrast to the traditional radial neck fracture and Monteggia fracture.

### 2.3. Operative technique

All patients were treated with closed reduction (CR) and plaster immobilization in the emergency room. Then, operation was performed on a radiolucent table after the induction of general anesthesia. The length of the ulna should be restored and stabilized according to the treatment principle of Monteggia fracture, and fixation techniques should depend on the location and type of ulna fracture. (1) For metaphysis fractures, patients with little displacement were left untreated or only manual reduction was done, while those with obvious displacement were fixed with smooth K-wires after reduction. (2) For proximal third ulna fractures, if the fracture was a short oblique fracture without longitudinal splitting, fixation was performed using an elastic stable intramedullary nail (ESIN). Generally, the diameter of the nail is approximately two-third of the isthmus diameter of the ulnar bone marrow, and it should be pre-bent into a C shape. (3) For long oblique fractures, open reduction (OR) and plate fixation were performed by using the posterior median approach. (4) For radial neck fractures, CR and retrograde ESIN fixation were performed with minimally invasive technology, and the tip of the nail was made to pass through the epiphyseal growth plate. If CR ended in failure in some instances, OR and smooth K-wire fixation was performed by adopting the anterior elbow Henry approach. After surgery, the arm was fixed in long arm plaster in a neutral position with 90° elbow flexion.

### 2.4. Assessments

Patients returned for a follow-up examination and radiographic evaluation under a protocol approved by the Ethics Committee. Anteroposterior and lateral radiographs of the entire ulna and radius were obtained to assess bony union, dislocation, ischemic necrosis of the radial head, early closure of the epiphysis, and heterotopic ossification. Clinical examination included an assessment of the rotation function and range of motion. When the x-ray shows a continuous callus passing through the fracture line, the plaster can be removed and functional exercise started under the doctor's guidance. As long as the fracture meets the clinical healing standard, the K-wires can be removed. For the ESIN and plate, the internal fixation can be removed only when an x-ray shows that the fracture line has disappeared completely and the medullary cavity is reopened. The therapeutic efficacy was evaluated at the final follow-up by using the Mayo Elbow Performance Score (MEPS) (5, 6).

## 3. Results

The 12 patients were followed up for 24–58 months (average, 40.5 months). The ulnar fractures were treated with CR and K-wire fixation in one patient, ESIN in four patients, OR and plate fixation in five, with no fixation in two. CR and ESIN proved successful in 11 patients with radial neck fracture, but one underwent OR and K-wire fixation (details in Table 1). All fractures healed on time without delayed union or non-union. Radial nerve injury occurred in one patient, and this patient recovered completely 3 months later. Avascular necrosis occurred in one patient and the bulk form of the proximal metaphysis manifested in another patient. The therapeutic efficacy was evaluated by using the MEPS, and it was found to be excellent in 10 patients, good in one, and fair in another.

**Table 1:** The details of patients and treatment results.

Case	Gender	Age	Type	Location of ulnar fracture	Location of radial neck fracture	Treatment of ulnar fracture	Treatment of radial neck fracture	Total follow-up (months)	Complication	MEPS
1	F	3	Extension-valgus	Proximal third	Metaphysis	CR + ESIN	CR + ESIN	36	No	Excellent
2	F	4	Extension-valgus	Proximal third	Metaphysis	CR + ESIN	CR + ESIN	42	No	Excellent
3	M	12	Extension-valgus	Middle third	Metaphysis	OR + Plate	CR + ESIN	54	No	Excellent
4	M	9	Flexion-varus	Proximal third	Metaphysis	OR + Plate	CR + ESIN	58	No	Excellent
5	F	4	Extension-valgus	Proximal third	Metaphysis	CR + ESIN	CR + ESIN	48	No	Excellent
6	F	5	Flexion-varus	Metaphysis	Metaphysis	No Treatment	CR + ESIN	36	No	Excellent
7	M	6	Extension-valgus	Metaphysis	Metaphysis	CR + K-wires	OR + K-wires	24	Avascular necrosis	Fair
8	F	8	Flexion-varus	Metaphysis	Metaphysis	CR	CR + ESIN	38	Bulk formation of proximal metaphysis	Good
9	M	12	Flexion-varus	Proximal third	Metaphysis	OR + Plate	CR + ESIN	46	No	Excellent
10	M	6	Extension-valgus	Middle third	Metaphysis	CR + ESIN	CR + ESIN	24	No	Excellent
11	M	7	Extension-valgus	Proximal third	Metaphysis	OR + Plate	CR + ESIN	50	No	Excellent
12	M	10	Flexion-varus	Proximal third	Metaphysis	OR + Plate	CR + ESIN	30	No	Excellent

CR, closed reduction; OR, open reduction; ESIN, elastic stable intramedullary nail.

## 4. Discussion

Monteggia fractures are rare injuries in children, accounting for only 5% of elbow fractures, mainly occurring in approximately 4- to 10-year olds (7). The Monteggia equivalent fractures proposed by Bado (1) refer to injuries that share similar mechanisms, imaging manifestations, and treatment principles with Monteggia fractures, mainly including Bado type I and type II, most of which do not lead to a separation of the proximal radioulnar joint, which is the main difference between the two fracture types. Five groups of type I equivalents were described: (Ia) anterior dislocation of the radial head; (Ib) fracture of the ulnar diaphysis with a fracture of the neck of the radius; (Ic) fracture of the neck of the radius; (Id) fracture of the ulnar diaphysis with a fracture of the proximal third of the radius; and (Ie) fracture of the ulnar diaphysis with an anterior dislocation of the radial head and a fracture of the olecranon. Type II equivalents were described: posterior radiocapitellar joint dislocation associated with epiphysis or radial neck fracture. The concept of “equivalent” for the pediatric population continued to evolve as further elucidation came from two study groups separately. Letts et al. (8) stressed the importance of noticing the anterior bend or greenstick of an immature ulnar and the subsequent dislocation or subluxation of the radiocapitellar joint in a pediatric Monteggia lesion. Letts and his colleagues took these occasions into the expanded equivalent lesions. Wiley and Galey (9) raised specific concerns on the olecranon and proximal ulnar fracture–related radiocapitellar joint issues. The authors suggested including the three scenarios in type I–III pediatric Monteggia equivalent lesions, respectively. Olney and Menelaus (10) and Čepelík et al. (11) proposed a classification based on the status of the radiocapitellar joint, as follows: group I: anterior ulnar plastic deformity combined with radial neck fracture and anterior radiocapitellar joint dislocation, group II: ulnar fracture associated with posterior radial neck fracture and posterior radiocapitellar joint dislocation, and group III: ulnar shaft fracture associated with a radial neck fracture. However, there are still disputes about the above classifications. Of course, with the increasing number of reported cases, some authors attempt to redefine or revise the concept and clinical classification of Monteggia equivalent fracture.

In our patients in this study, this special type of Monteggia equivalent fracture was characterized by an ulna fracture, located in the middle and above (metaphysis, proximal third, middle third), most of which had obvious angulation. The accompanying radial neck fractures were located in the metaphysis, without involving the epiphysis and epiphyseal plate. This was in total contrast to the traditional radial neck fracture. In addition, the lesion was characterized by a separated fracture of the radial neck with no dislocation of the radial head. A line appeared through the longitudinal axis of the radius off the center of the capitellum, but with an intact annular ligament and normal radiocapitellar line, which was different from the Monteggia fracture. This type of injury is very rare, perhaps caused by a relative relaxation of the annular ligament in children, so we called it “head-neck separation type.” The relevant literature is very rare, and all are case reports, so we reported a total of 12 cases, the largest number to date.

Our classification was based on the direction of displacement and angulation of fractures on radiographs, including the extension-valgus type and flexion-varus type. We concluded that the characteristic of the extension-valgus type was an ulnar fracture with volar ulnar angulation plus a radial neck fracture with a volar ulnar displacement of the distal end, which was similar to the mechanism of type I Monteggia fracture. When falling is supported by the palm, the forearm is in the extension supination position, and the stress is transmitted upward along the forearm. The radial neck fracture is first caused by the vertical and valgus stress. The strong contraction of the biceps muscle caused by elbow hyperextension makes the distal end of the radial fracture at the attachment point shift to the proximal and volar side, resulting in a complete separation of the head and neck. The stress of hyperextension and valgus continues to transmit, resulting in an ulna fracture with volar ulnar angulation. However, the flexion-varus type is opposite to the former, with angulation and displacement direction of the fracture point to the radial dorsal side. The injury mechanism is similar to type II Monteggia fracture. When falling is supported by the palm, the elbow is in a semiflexion position and pronation position, resulting in an ulnar fracture under the combined action of axial and varus stresses. At the same time, the proximal radius strikes the capitulum of the humerus, resulting in a radial neck fracture, while the proximal end of the radial neck fracture is located in the joint capsule, and the distal end of the fracture point is on the radial dorsal side. In our report, seven cases belonged to the extension-valgus type, and five cases were classified as the flexion-varus type. Compared with the three cases reported in the previous literature, all of them were of the extension-valgus type.

The majority of fresh Monteggia fractures in children can yield satisfactory results by performing CR and plaster immobilization. In contrast, most Monteggia equivalent fractures in children require surgical treatment; otherwise, the prognosis is considered poor (12, 13). Anatomic reduction of the proximal radius is essential for its function. However, if the reduction is unsatisfactory or redisplaced, the prognosis would be affected, and therefore, surgical treatment is recommended (12, 13). In our patients in this study, if CR and plaster immobilization are performed for extension-valgus-type fractures, the forearm needs to be fixed in the position of supination with extreme elbow flexion (100–110°). The greater the flexion angle is, the more stable the reduction will be, but this will increase the risk of forearm osteofascial compartment syndrome. For flexion-varus-type fractures, elbow extension with plaster fixation is required, which obviously limits daily life activities combined with the inconveniences resulting from nursing. At the same time, the fracture may be displaced again. Therefore, all patients were treated surgically in our department.

**Figure 3:**
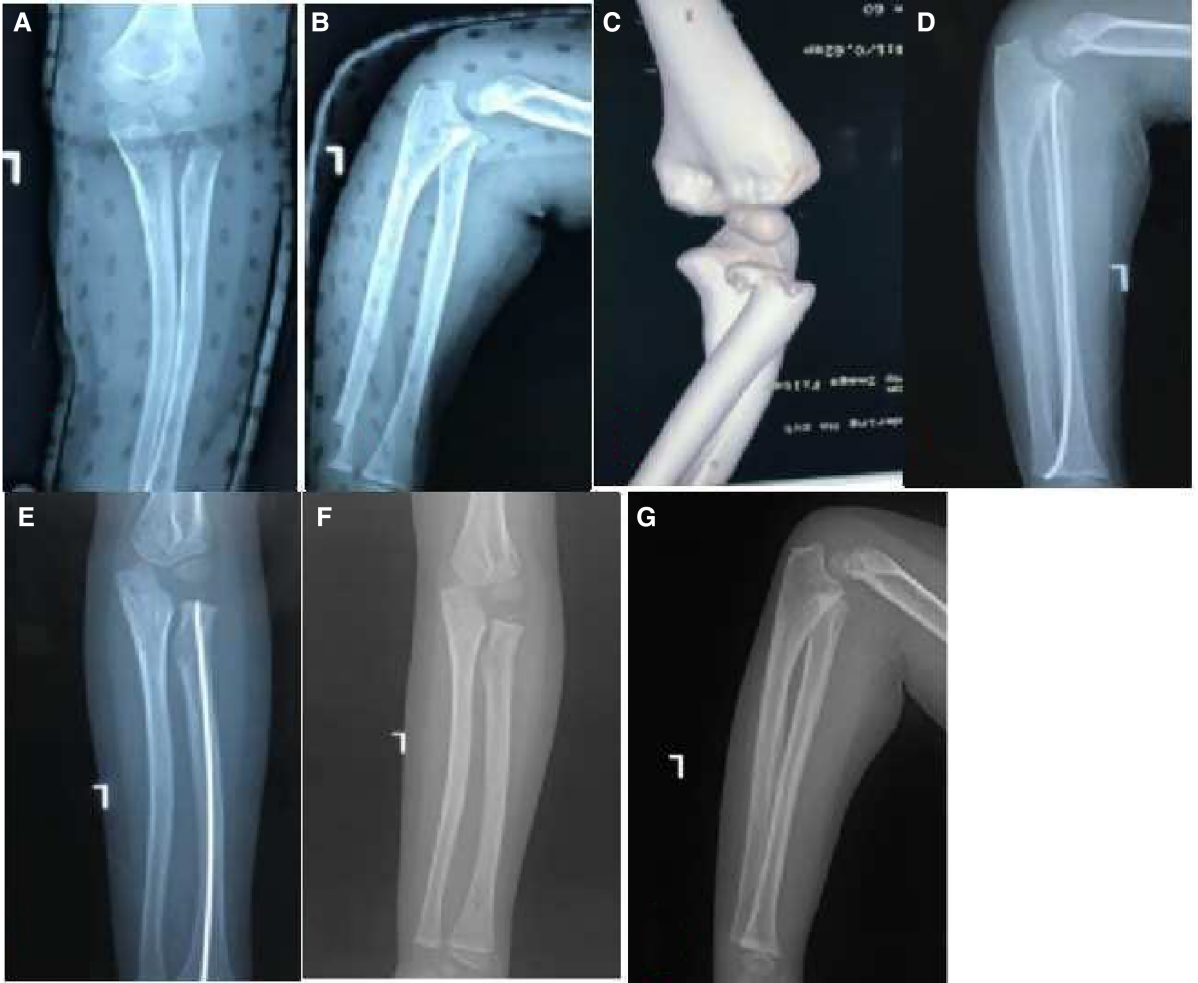
A 5-year-old girl with a head–neck separation type of Monteggia equivalent fractures (flexion-varus type) (**A–C**). No treatment for ulna greenstick fracture, closed reduction with elastic intramedullary nail for radial neck fracture (**D,E**). The last follow-up was 3 years after surgery (**F,G**).

**Figure 4:**
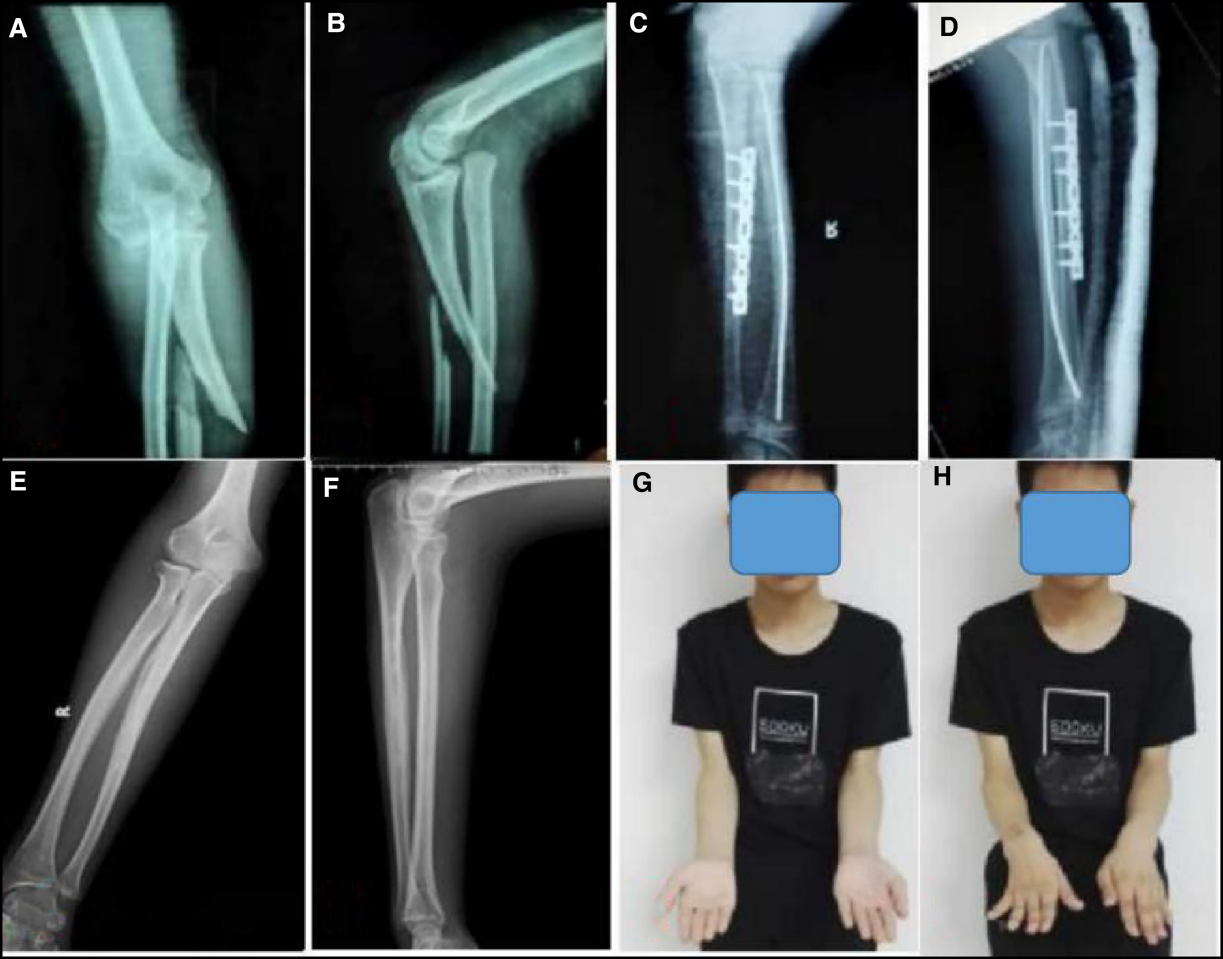
A 12-year-old boy with a head–neck separation type of Monteggia equivalent fractures (extension-valgus type) (**A,B**). OR and internal fixation with a bone plate for an ulna fracture and CR with ESIN for a radial neck fracture (**C,D**). The last follow-up was 4.5 years after surgery, which showed normal elbow movement and an excellent MEPS (**E–H**).

The treatment principle is similar to that for Monteggia fracture. First, restore and stabilize the length of the ulna and then deal with the radial neck fracture. In this report, one patient with ulna metaphysis fracture with little displacement did not require treatment, one patient with greenstick fracture underwent manual reduction (Figure 3), and the rest underwent CR and K-wire fixation. For the proximal and middle third ulna fractures, ESIN is minimally invasive and does not require cutting of the fracture end; also there is no loss of blood supply and there are relatively few complications (14). It is suitable for short oblique fractures of the ulna, but the rate of stability is poor for long oblique fractures. In patients with proximal third ulna fractures, if the proximal end of the fracture has a longitudinal splitting and the proximal ulna has a relatively wide medullary cavity, the insertion point of the nail will be closer to the fracture line, which makes it difficult to achieve a three-point fixation and is not conducive to reduction and stability. In such patients, plate fixation may be more ideal. Therefore, in our seven patients with proximal third ulna fractures, four were treated with plate fixation and three with ESIN fixation. In two patients with middle third ulna fractures, one (short oblique type) was treated with CR and ESIN, and the other (long oblique type) was treated with OR and plate fixation (Figure 4).

Although the treatment of radial neck fractures remains controversial, most authors still prefer minimally invasive treatment. OR can lead to complications such as early closure of the proximal radius epiphysis, a long radial head, ischemic necrosis of the radial head epiphysis, and elbow dysfunction (15). According to a retrospective analysis by Basmajian et al. (16), the success rate of percutaneous minimally invasive treatment of radial neck fractures is 73%, while that of OR is only 35%. According to an analysis by Yilmaz, the MEPS in the Métaizeau technique group was 95.2, with excellent results in 15 patients (68%), good results in seven (31%), and fair or poor results in none of the patients, but the mean MEPS in the open reduction/K-wire group was 88, with excellent, good, fair, and poor results in nine (36%), 12 (48%), four (16%), and none of the patients, respectively (17). In our patients, after satisfactory reduction and fixation of the ulna fracture, we attempted to perform CR and a retrograde ESIN for radial neck fracture, and we succeeded in 11 patients. In order to avoid re-displacement, the tip of the nail was passed through the epiphyseal plate to increase stability. One patient underwent OR and K-wire fixation because of the failure of CR. During the operation, the fracture line was found to be located outside the joint capsule, with a separation of the head and neck and obvious displacement, but the proximal position of the fracture and proximal radioulnar joint were normal and the annular ligament was intact, which also confirmed our previous inference and injury mechanism. The therapeutic efficacy was evaluated at the final follow-up by using the MEPS and it was found to be excellent in 10 patients, good in one, and fair in another.

## 5. Limitations

There are several limitations in our study. First, this is a retrospective analysis, and the number of cases is small, and therefore, we were not able to carry out a statistical comparative analysis. In addition, the follow-up time of some patients was short, and as a consequence, whether there would be later development of complications is difficult to predict.

## 6. Conclusion

The head–neck separation type of Monteggia equivalent fractures in children is rare. Its clinical characteristics are different from those of Monteggia fracture and radial neck fracture. According to the location and type of ulna fracture, the length and anatomic position of the ulna should be restored and stabilized first, while the radial neck fracture should be treated with CR and ESIN fixation. Through such standard treatment and early functional exercise, satisfactory clinical results can be achieved.

## Data Availability

The original contributions presented in the study are included in the article/Supplementary Material; further inquiries can be directed to the corresponding authors.
